# Editorial for Special Issue “Unleashing the Hidden Potential of Anaerobic Fungi”

**DOI:** 10.3390/microorganisms11030652

**Published:** 2023-03-03

**Authors:** Michael Lebuhn, Sabine Marie Podmirseg, Urs Baier

**Affiliations:** 1 Department for Quality Assurance and Analytics, Bavarian State Research Center for Agriculture, 85354 Freising, Germany; 2 Department of Microbiology, Universität Innsbruck, Innrain 52, 6020 Innsbruck, Austria; 3 Institute of Chemistry and Biotechnology (ICBT), Zurich University of Applied Sciences (ZHAW), 8820 Wädenswil, Switzerland

Anaerobic fungi (AF) of the phylum *Neocallimastigomycota* are a very peculiar group of microorganisms. Since their first discovery in the early nineteen-hundreds and assignment to the kingdom *Fungi* in 1975 by Orpin [1], many researchers have delved into these highly potent degraders of lignocellulosic biomass (LCB). Their panoply of hydrolytic enzymes makes them key players in the digestive tract of herbivores, but their occurrence may not be restricted to this habitat alone. Despite the plenitude of research on this AF group, many questions still remain unanswered, and the implementation of AF within, e.g., biomethanation of LCB or bioethanol production, is still in its infancy. This is where international projects such as “Unleashing the Hidden Potential of Anaerobic Fungi” (https://www.hipoaf.com; accessed last on 14 February 2023) hook in and aim at answering basic questions such as ideal growth conditions, improved and novel detection techniques, screening for novel habitats, strains and enzymes, symbiotic interactions of AF, and, eventually, at paving the way to the successful biotechnological implementation of these unique microorganisms. These questions are also the scope of this Special Issue, comprising eight original articles and two reviews that are dedicated to recent updates on various fields of AF research. The contributions span from AF in animal husbandry and biotechnology over AF systematics, physiology and molecular detection to isolation strategies. The highlights of these works are introduced here briefly:

Phylogenetic AF lineages, for whose existence only molecular biological data were available so far, could be cultivated successively. Stabel et al. [2] present the remarkable example of *Aestipascuomyces dupliciliberans*, a species with transcontinental distribution that has been isolated in parallel from digestive tracts of sheep and alpaca. This species was identified as the former SK4 clade that has been known only from molecular analyses.

Major cognition progress on the existence of *Neocallimastigomycetes* in unexplored habitats is expected from application of the novel *Neocallimastigomycetes*-specific primer pair targeting the D2 region of the large ribosomal subunit, which is described in the article of Young et al. [3], and can be used simultaneously for quantification and phylogenetic analysis. With these primers, the authors identified several hitherto unknown sequence clusters, among these the huge YL2 (“Mara”) clade from mara feces for which cultivation trials have already been initiated. Further, filling a major knowledge gap is expected from applying the primers on potential habitats of *Neocallimastigomycetes* outside the digestive tract of herbivores.

Fliegerová et al. [4] demonstrate that upon diet change from alfalfa hay to a high grain diet, considerable changes in the relative abundance of AF genera occur in goat rumen. Still, the initial community composition of each individual host seems decisive in the establishing species pattern. Furthermore, this molecular monitoring study points out that abundantly investigated host animals such as goats, might still act as reservoirs for novel or yet uncultured isolates such as the BlackRhino group.

In a related study, monitoring the diet shift from hay to alfalfa for cattle, Azad and co-workers also observed a major change within the rumen AF community [5]. The focus was set on the emergence of frothy bloat, a digestive disorder commonly associated with an alfalfa diet. This disease pattern was not accompanied with a specific AF community pattern, but with a potential disruption of the normal rumen microbiota, as indicated by network analyses between AF and rumen bacteria.

As cultivation is central to any taxonomic description and many biotechnological applications but known to be challenging, especially for AF, significant progress can be expected from respective improvements. Using the primers of Young et al., Joshi et al. [6] show how the presence of rumen fluid in the medium influences the isolate spectrum, and that its omission favors the enrichment of different strains. The authors conclude that the medium composition should mimic the natural habitat as close as possible if the “true” original composition of *Neocallimastigomycetes* is being examined.

Another article on the isolation of AF from zoo animal feces comprehensively characterizes the biochemical spectrum of six strains grown on different carbon sources. Here, Stabel et al. [7] demonstrate the consistent preference for hydrogen, acetate and formate production by AF as core metabolites, and more strain-specific generation of succinate and lactate. Moreover, this study also suggests the regrouping of *Cyllamyces* into the *Caecomyces* clade, challenging actual phylogenetic presentations.

Already in the early 1980s, a high potential of AF for biofuels production from LCB was postulated, as Saye et al. explain in their comprehensive review [8]. Understanding their life cycle and the ecological niche that AF colonize are prerequisites for successful application on a technical scale. For the production of biofuels, H_2_ and platform chemicals, consolidated bioprocesses are described to be more promising than enzymatic pre-treatments. In addition to the need for robust cultivation and genetic tools for AF, the lack of scalable bioreactor systems that simulate the natural environment of the ruminant digestive tract is identified as a bottleneck for industrial use.

This limitation is also tackled by the work of Jimenez and colleagues [9] on substrate pre-treatment. The authors were able to reduce the lag-phase of AF growth and to improve the fermentation product yield. Their work represents another step forward towards stable and predictable AF bioprocesses.

Describing new fields for biotechnological applications of AF also means deep comprehension of their metabolic capabilities and interrelations with other microbial groups such as methanogens. Li et al. [10] exhaustively review the connectivity of these two groups and summarize the state of the art and actual know-how in this field. The co-cultivation of both key players is suggested as an optimal approach to improve the overall performance in biomethanation of LCB.

Finally, with respect to the lack of genetic tools, Hillman and his co-authors [11] identified the AT-richness of AF genomes being a major obstacle for the heterologous expression of AF pathways in model hosts. Their findings open new doors to the exploitation of the biosynthetic potential of AF via codon usage and optimization.

We are pleased to present novel discoveries in this wide range of properties and potentials of AF to the readership of *Microorganisms*. Many developments are in their infancy, and many questions remain to be resolved. However, as these peculiar microorganisms are attracting more and more worldwide attention, the relevant perceptions, insights and handling expertise will significantly increase soon.
